# Is It Time for a Thai Cancer Drug Fund? From Global Evidence to Collaborative Action – A Response to Recent Commentary

**DOI:** 10.34172/ijhpm.10127

**Published:** 2026-07-22

**Authors:** Parnnaphat Luksameesate, Osot Nerapusee, Chanthawat Patikorn, Puree Anantachoti

**Affiliations:** ^1^Social and Administrative Pharmacy Department, Faculty of Pharmaceutical Sciences, Chulalongkorn University, Bangkok, Thailand.; ^2^School of Pharmacy, Eastern Asia University, Pathum Thani, Thailand.

 We would like to thank Dr. Jong Hyuk Lee for his insightful commentary on our study, “Scoping Review of International Experience of a Dedicated Fund to Support Patient Access to Cancer Drugs: Policy Implications for Thailand.”^1^ His highlights on balancing innovation, affordability, and equity provide an invaluable extension to our policy recommendations and open up crucial pathways for structural refinement.^2^ Below, we address the specific methodological points raised and further contextualize the operational, social, and fiscal dimensions within Thailand’s current healthcare landscape.

## Addressing Methodological and Data Points

 We highly appreciate Lee’s thorough review of the data. Regarding the specific figures, such as the number of covered cancer drugs under England’s Cancer Drugs Fund (CDF) standing at 102 instead of 95, we acknowledge that his data reflected the updated status at that specific time point. The figures presented in our original article were bounded by the data collection timeframe of 2022-2023. Furthermore, we agree with Lee’s assertion regarding the importance of local-language official documents and grey literature in capturing nuanced regulatory mechanisms. Our review indeed incorporated both published and grey literature to minimize translation and interpretation biases across jurisdictions.

## The Thai Context: Fragmentation and Unmet Medical Needs

 To understand the necessity of a ring-fenced CDF and managed entry agreements in Thailand, the structural characteristics of its healthcare system must be examined. Nominally, 100% of the Thai population has been covered under three major public health insurance schemes since 2002: the universal coverage scheme, the social security scheme, and the civil servant medical benefit scheme.^3^ Patients incur no out-of-pocket costs for medications listed within the core benefits package. While all three schemes share the same National List of Essential Medicines (NLEM) as their baseline, their historical origins differ, allowing each scheme to independently expand certain benefits. For instance, the Civil Servant Medical Benefit Scheme operates the Outpatient Cancer Prior Authorization program allow expansion of certain cancer drugs under restricted conditions.^4^

 Despite these provisions, a significant policy gap persists. Numerous innovative medicines remain excluded from the NLEM, often taking several years (until generics or stand-alone biologics emerge) to be integrated.^5^ Oncology and rare diseases represent the areas with the highest unmet medical needs, where high treatment costs expose citizens to catastrophic health expenditure.^6^ The NLEM relies strictly on health technology assessment (HTA) frameworks, applying an incremental cost-effectiveness ratio threshold of 160 000 Baht per quality-adjusted life-year alongside rigorous budget impact analyses. Consequently, innovative cancer therapies—with incremental cost-effectiveness ratios exponentially exceeding this threshold—are structurally blocked from routine reimbursement, leaving patients financially vulnerable.^4,7^

## Stakeholder Consensus and Operational Governance

 As researchers, we argue for a philosophical pivot in insurance design, moving toward protecting against catastrophic illness, establishing shared responsibilities, and introducing complementary mechanisms, such as a CDF, to safeguard access to life-saving treatments without replacing Thailand’s existing HTA and NLEM processes. Following our scoping review, we presented international evidence on CDFs and managed entry agreements to key stakeholders, culminating in stakeholder consensus meetings to explore local feasibility.

 The majority of stakeholders agreed that a Thai CDF is feasible but demanded absolute clarity on several operational parameters to guarantee evidence-based, fair, and transparent implementation^8^:

1. Equity across schemes: The CDF must provide uniform access across all three public health insurance schemes to eliminate disparities.
2. Targeted scope: The fund must explicitly prioritize life-saving and practice-changing medicines that fail standard NLEM approval due to lack of cost-effectiveness or excessive budget impact.
3. Optimized selection process: To minimize administrative delays, the drug selection process should seamlessly follow the NLEM evaluation pipeline. If a drug fails the routine HTA pathway, it should be immediately screened for CDF eligibility based on refined criteria, which need to be developed and explored their practicality in Thailand.
4. Governance and infrastructure: Developing explicit CDF budget estimation models, public accountability mechanisms, and robust real-world evidence infrastructure is vital for monitoring and evaluation. Legal and regulatory frameworks must also be amended to accommodate manufacturer rebates.

## Public Perceptions and Future Policy Directions (2025–2027)

 To address the critical attribute of “shared responsibility” and understand public tolerance toward financial risk-pooling, our research team conducted a qualitative study between 2025 and 2026. Through in-depth interviews across beneficiaries of all three insurance schemes spanning four distinct household income levels, we made a striking finding: Thai citizens across all socio-economic strata expressed a willingness to share financial responsibility for minor illnesses, chronic diseases, and even severe conditions, provided that robust safety nets are established. However, their actual financial capacity varies significantly by income and disease severity.

 Moving forward, our team has initiated the development of explicit drug selection criteria tailored for the Thai CDF. This collaborative refinement process with clinical experts and policy stakeholders is projected for completion in 2027. Amid tight national fiscal constraints, our framework recommends that the CDF prioritize drugs with robust evidence of meaningful clinical benefit while ensuring a transparent and evidence-based selection process. Operationally, the CDF should primarily focus on improving timely patient access.

## Conclusion

 A well-structured, managed-access fund must align seamlessly with Thailand’s socio-economic realities. The continuous academic exchange between our findings and his commentary serves as a crucial cornerstone for policy-makers, bridging the gap among innovation, affordability, and equity in cancer care.

## Disclosure of artificial intelligence (AI) use

- Section(s) of manuscript affected: The entire manuscript (overall language editing and proofreading).
- Type of AI tool(s) used: Gemini (Google).
- Purpose of use: AI was strictly only use for language editing, grammatical corrections, and proofreading to improve clarity and readability.

## Ethical issues

 Not applicable.

## Conflicts of interest

 Authors declare that they have no conflicts of interest.

## Disclaimer

 The opinions expressed in this paper are authors’s opinion and do not represent the views of their institutions or other stakeholders.
